# New species of *Goniocolletes* and *Trichocolletes* (Hymenoptera, Colletidae) from southern Australia

**DOI:** 10.3897/zookeys.598.9229

**Published:** 2016-06-14

**Authors:** Remko Leijs, Katja Hogendoorn

**Affiliations:** 1 South Australian Museum, North Terrace, Adelaide, SA 5000; 2 School of Agriculture, Food and Wine, The University of Adelaide, SA 5005

**Keywords:** Colletid bees, Bush Blitz survey

## Abstract

*Goniocolletes comatus* Maynard, 2013 is redescribed. *Goniocolletes wanni* **sp. n.** and the male of *Trichocolletes luteorufus* Batley & Houston, 2012 are described.

## Introduction

This paper reports on bee species that were collected on Bush Blitz surveys in remote locations of Australia. Bush Blitz is a partnership between the Australian Government, BHP Billiton and Earthwatch Australia to document fauna and flora on selected national reserves. These surveys regularly result in the discovery of new invertebrate species (e.g. true bugs: Symonds and Cassis 2014; spiders: Baehr et al. 2013, bees: Hogendoorn et al. 2015).

The Australian native bee species are still relatively unknown. Close to 60% of the known species are not in identification keys and with each generic revision numerous new species are added (Batley and Hogendoorn 2009). Therefore, species identification and recognition of new species is most straightforward for those genera that have recently been revised and where species identification keys have been included in the revision (Hogendoorn et al 2015). Here, we describe species in two genera of Colletidae that were revised recently (*Trichocolletes*: Batley and Houston 2012; *Goniocolletes*: Maynard 2013). The descriptions cover related genera and involve one new species, one redescription and a description of a male for a species of which hitherto only the female was known.

## Methods

Bee species were caught on flowering plants using a hand net. Specimens of *Trichocolletes* and *Goniocolletes* were identified using identification keys in recent revisions of these genera by Batley and Houston (2012) and Maynard (2013) respectively. The identified specimens were compared to type specimens and other reliably named material at the Queensland Museum, the Western Australian Museum and the South Australian Museum.

For descriptions of the new species the terminology used by Michener (2007) was followed. Using a stereomicroscope with step-less zoom and an eyepiece micro-meter measurements were taken relative to the head width, whereby head width was set to 50 units (following Houston 1990). Abbreviations for these relative measurements are as follows: AOD antennocular distance; ASD antennal socket diameter; BMW basal width of mandible; DMA distance between anterior mandibular articulations; FL flagellum length; HL head length; HVO height of vertex above lateral ocelli; HW head width; IAD interantennal distance; LID lower interorbital distance; ML mandible length; MOD diameter of median ocellus; MSL malar space length; OOD ocellocular distance; SL scape length; SW scape width; UFW upper width of face; UID upper interorbital distance; WOC width of ocellar cluster. Other abbreviations used are: T1, T2 etc. for first, second metasomal tergite; S1, S2 for first, second metasomal sternite, etc.

Some of the specimens treated here were also submitted to BOLD (Barcode of Life Database) for DNA barcoding using the cytochrome c oxidase subunit 1 gene. Specimen details, including DNA sequence, collecting dates and locality information can be accessed in BOLD under the project Australian Bee Survey, e.g. http://www.boldsystems.org/index.php/Public_RecordView?processid=AUSBS310-13

AUSBS-numbers are presented under material examined.

Under material examined, collection data are presented as copied from the specimen labels. Therefore, data formats for locality coordinates could vary. For specimens collected by Leijs et al. the locality coordinates are in decimal degrees.

### Repositories

ANIC
Australian National Insect Collection



SAMA
 South Australian Museum, Adelaide 


QM
Queensland Museum, which now also contains the former collections of the University of Queensland Insect Collection (UQIC) 


## Systematics

### Goniocolletes

Taxon classificationAnimaliaHymenopteraColletidae

Cockerell, 1907

#### Remarks.

Two species of *Goniocolletes* were collected during a Bush Blitz survey at Hiltaba Station circa 130 km East of Ceduna, South Australia, November 2012. A single specimen of *Goniocolletes abdominalis* (SAMA32-032978 / AUSBS 313-13) was collected on the same flowering *Eucalyptus* as where a number of other *Goniocolletes* males were caught. Using the key and information provided in Maynard’s (2013) revision, these other collected specimens of *Goniocolletes* initially keyed out to *Goniocolletes comatus* Maynard 2013. However, unlike the colour characters mentioned in Maynard (2013), our specimens had orange legs, with dark coxae and trochanters. Comparison to the type specimen showed that these colour characters seem to have been switched both in the identification key (Maynard 2013, pg. 98) and species description (Maynard 2013, pg. 104). Locating the holotype of *Goniocolletes comatus* and other species examined specimens by Maynard (2013) was not straightforward, because none of the type specimens in SAMA and QM had been labelled as such. The male (holo)-type of *Goniocolletes comatus* could be identified and located on the basis of its unique locality data, but this was impossible for the female type, because multiple specimens had the same label information as the type (Maynard 2013), and their repository was not stated. Examination of all specimens associated with *Goniocolletes comatus* in the collections of QM and SAMA, indicated the existence of two species, with differences in pubescence and characters associated with the male genitalia and hidden sternites. We associated the sexes by using series of males and females collected from the same date and locality for each of the species. Here, we redescribe the male and female of *Goniocolletes comatus* and describe the additional species on the basis of fresh material that was collected during a Bush Blitz survey at Hiltaba Station, South Australia.

As only a single species is added to this genus, we do not produce a completely new key, but suggest modifying the key produced by Maynard (2013, pg. 97) as follows:

For males:

**Table:** 

9(8)	Legs all black	***Goniocolletes albopilosus* (Rayment)**
–	Legs orange with black coxae and trochanters	**9a**
9a(9)	Pubescence on T1 not dense and less than twice as long as on T2 (Fig. 1A, 1B), tip of S8 with long hairs pointed backwards (Fig. 3D)	***Goniocolletes comatus* Maynard**
–	Pubescence on T1 dense and more than twice as long as on T2 (Fig. 2A, 2B), tip of S8 with a sidewards directed patch of dense stiff long dark hairs on each side (Fig. 4D)	***Goniocolletes wanni* sp. n.**

**Figure 1, 2.**
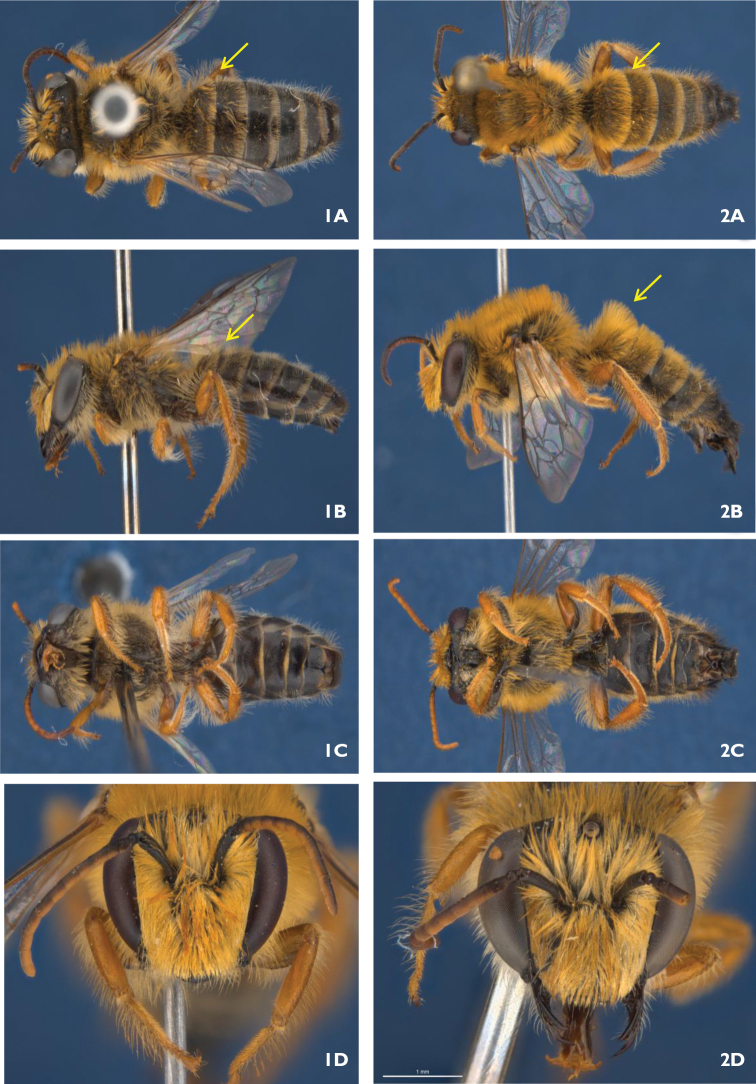
**1** Male holotype *Goniocolletes comatus* (SAMA 32-032610): **A** dorsal **B** lateral **C** ventral **D** head **2** Male holotype *Goniocolletes wanni* sp. n. (SAMA 32-032979): **A** dorsal **B** lateral **C** ventral **D** head.

**Figure 3.**
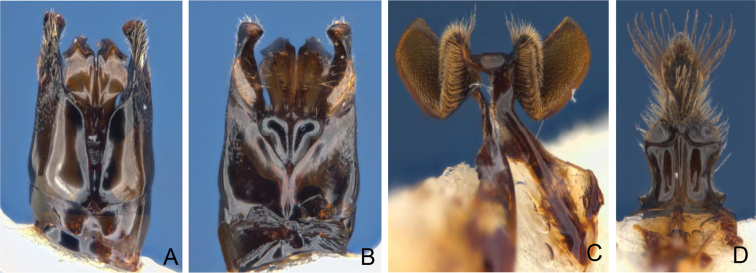
Male holotype *Goniocolletes comatus* (SAMA 32-032610). **A** genital capsule dorsal **B** id. ventral **C** S7 **D** S8.

**Figure 4.**
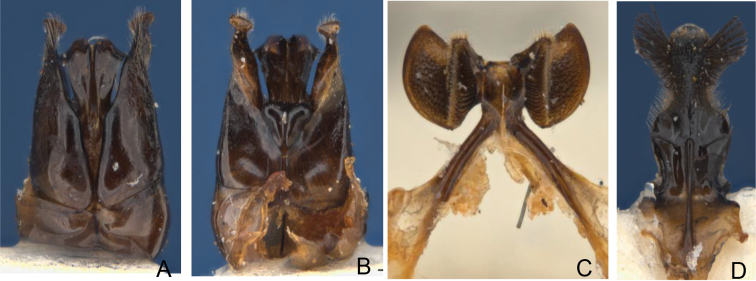
Male holotype *Goniocolletes wanni* sp. n. (SAMA 32-032979). **A** genital capsule dorsal **B** id. ventral **C** S7 **D** S8.

For females:

**Table:** 

24(23)	Median area of supraclypeal area punctate	***Goniocolletes anthedonus* Maynard**
–	Median area of supraclypeal area polished and impunctate	**25**
25(24)	Horizontal part of propodeal triangle gradually sloping, first flagellomere similar in length to third	***Goniocolletes comatus* Maynard**
–	Horizontal part of propodeal triangle defined by sharp carinae, first flagellomere longer than third	***Goniocolletes wanni* sp. n.**

### Goniocolletes comatus

Taxon classificationAnimaliaHymenopteraColletidae

Maynard, 2013

Figs 1 , 3 , 5

#### Material examined.

*Holotype*: ♂, Lake Gilles NP, 3 Feb 1975, C.A. & T.F. Houston, on *Eucalyptus* blossom (SAMA 32-032610).

#### Specimen used for the description of the female.

♀, Lake Gilles NP, SA, 8 Mar 1976, C.A. & T.F. Houston, on *Eucalyptus* (SAMA 32-033291).

#### Additional material examined

17♂. **SAMA**: 4♂, Lake Gilles NP, SA, 8 Mar 1976, C.A. & T.F. Houston, on *Eucalyptus*;

1♂, Orroroo, SA, 22 Apr 1944. **ANIC**: 10♂, Angorichina Hotel, 31.08S 138.34E, SA, 8 Nov 1987, I. Nauman & J. Cardale, on *Eucalypt* flowers; 2♂, Brachina, 31.01S 138.33E, 9 Nov 1987, I. Nauman & J. Cardale.

#### Diagnosis.

This species is distinguished from other species in this genus by males with black metasoma, simple orange legs with brown coxae and trochanters, scape black, pubescence on T1 as long as on T2 and S8 with evenly and openly placed long radiating setae. Females with propodeal triangle gradually sloping downwards, horizontal area not defined by a sharp carina.

#### Redescriptions.

*Holotype*: Male (SAMA 32-032610), body length 8.8 mm, head width 2.6 mm.

*Relative head measurements*. HW 50, HL 42, UID 30, UFW 33, LID 27, DMA 28, HVO 0, WOC 15, MOD 4, OOD 8, IAD 8, ASD 3, AOD 7, ML 21, BMW 8, MSL 1, SL 13, SW 3, FL 52.

*Structure*. Head: face longer than wide, clearly converging below; malar space circa 0.13× basal mandibular width; clypeus protuberant; vertex not elevated; gena about 0.8× eye width viewed laterally; flagellum as long as head width; flagellomeres 3-11 1.4× as long as wide. Legs not modified: hind tarsus longer than hind tibia; hind basitarsus 7× as long as wide; hind coxa without posterior ventral procession; S7 (Fig. 3C), inner lobes almost straight broadly rounded proximally; S8 (Fig. 3D) with evenly and openly placed long radiating setae, apparent in un-dissected specimens; genital capsule (Fig. 3AB), penis valves suddenly broadened at level of mid gonocoxite.

*Coloration*. Integument black, apart from antenna ventrally brown, legs orange, apart from basis of femur of front and middle legs, all coxae and trochanters brown, tegula translucent orange; marginal zone of T1-6 transparent.

*Sculpture*. Scutum, propodeum and tergites with close pit-reticulation, vertical part of propodeal triangle dull with fine irregular reticulation, horizontal part with transverse striae, lateral rim coarsely areolate.

*Pubescence*. Eyes with tiny dispersed hairs. Pubescence orange, apart from posterior margin of T6 and whole of T6-7 black, genae near eye margin whitish. Hairs on face erect, dense and long; hairs on T1 longer than on T2-4; sternites almost bare, apart from S3-4 with narrow, dense posterior fringes.

Female (SAMA 32-033291), body length circa 11 mm, head width 3.2 mm.

*Relative head measurements*. HW 50, HL 40, UID 28, UFW 34, LID 29, DMA 27, HVO 1, WOC 16, MOD 4, OOD 6, IAD 8, ASD 3, AOD 8, ML 21, BMW 7, MSL 2, SL 15, SW 3, FL 33.

*Structure*: Head: inner eye margin straight, converging below; scape reaching median ocellus, tapering basally; malar space smooth length about 0.16 width; labial palps and maxillary palps just reaching apex of extended glossa; clypeus below supraclypeal level; epistomal suture distinct, almost straight; labrum basally strongly produced, apically flat; F1,3–10 length equal to, or less than width, F2 shorter than width; antennal sockets depressed; supraclypeal area strongly raised above frons level; midtibial spur long, slender; inner hind tibial spur with 6 long, slender teeth (other examined specimens with 7 teeth); Metasoma: postgradular area with weaker sculpture than pregradular area; pygidial plate broadly rounded apically, flat, dull; T2-4 anteriorly fine reticulate striated, posteriorly open to close punctated.

*Coloration*: Head, integument black with dark brown to black clypeus; mandibles black, medially brown and smooth; scape almost naked, black; flagellum light brown beneath, dark brown above; Mesosomal integument black; Metasomal integument orange.

*Sculpture*: Scutum and scutellum posteriomedially smooth with strong open punctation; metanotum dull and rugose; propodeal triangle with several fine transverse carina, anterior area narrower than metanotum; anterior area almost smooth. Clypeus flat, shiny with open large punctures; supraclypeal area polished, flat.

*Pubescence*: Head: hair white, dense on lower paraocular area; mesosomal hair white; hind basitibial plate with dense, branched hairs, obscuring marginal carina; hind basitarsus with posterior open fringe of long, branched grey hairs. Metasomal hair dorsally short, sparse, white; prepygidial fimbria dark brown; T2-4 posteriorly with fringes of long white hair.

#### Remarks.

A redescription of this species was necessary because the specimens examined under Maynard’s (2013) original description of *Goniocolletes comatus*, appear to belong to more than one species. The male holotype and the female are redescribed for comparative reasons.

#### Floral visitations.

*Eucalyptus* (Myrtaceae)

#### Distribution.

Currently known from four localities in the Flinders Ranges and northern Eyre Peninsula, South Australia.

**Figure 5, 6.**
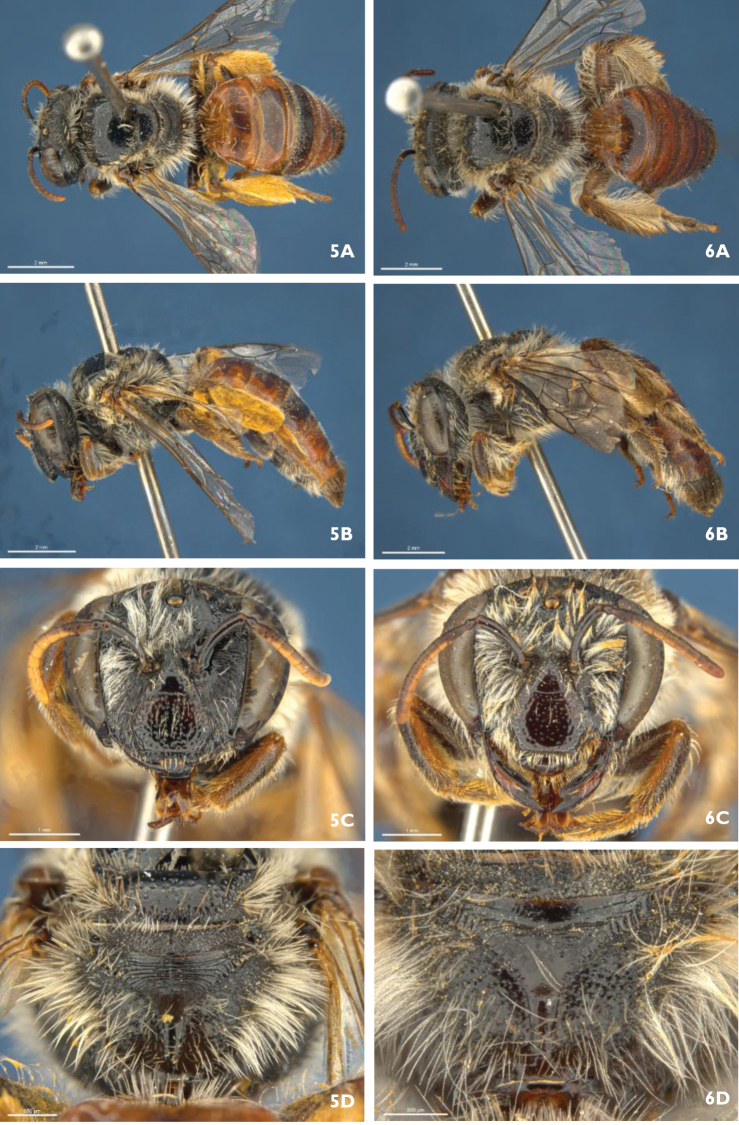
**5** Female *Goniocolletes comatus* (SAMA 32-033291): **A** dorsal **B** lateral **C** head **D** propodeum **6** Female paratype *Goniocolletes wanni* sp. n. (SAMA 32-033292): **A** dorsal **B** lateral **C** head **D** propodeum.

### Goniocolletes wanni sp. n.

Taxon classificationAnimaliaHymenopteraColletidae

http://zoobank.org/E2A89228-CB9B-46B0-A07A-98CB0A5804D2

Figs 2 , 4 , 6

#### Material examined.

*Holotype*: ♂, (SAMA32-032979-AUSBS310-13), Hiltaba Station, SA, 32.11689S 135.15634E, 16 Nov 2012, R. Leijs and B. Tully, on *Eucalyptus* (SAMA).

#### Paratype.

♀, (SAMA 32-033292), 11 miles (17.6 km) S of Salmon Gums, WA, 11 Jan 1970, T.F. Houston, on *Eucalyptus*.

#### Additional material examined

5♀, 42♂. **SAMA**: 1♂, (SAMA 32-033115), Hiltaba Station, SA, 32.12293S 135.22875E, 12 Nov 2012, R. Leijs and B. Tully, on *Myoporum*; 3♂, (SAMA32-033276-AUSBS 312-13, SAMA32-033277-AUSBS 311-13, SAMA32-033277-AUSBS 311-13), Hiltaba Station, N of Progress Dam, SA, 32.23577S, 135.26811E, 12 Nov 2012, R. Leijs and B. Tully, on *Eucalyptus*; 1♂ 28 km NE Wirrulla, SA, 7 Mar 1976, C.A. & T.F. Houston, on *Eucalyptus*; 1♂ 20 miles (32km) NE Eucla, SA, 9 Jan 1970, T.F. Houston; 1♀, 3♂ Moorlands, SA, 19 Jan 1966, T.F. Houston on *Eucalyptus*. SA: 5♀, 2♂ 11 miles (17.6km) S of Salmon Gums, WA, 11 Jan 1970, T.F. Houston, on *Eucalyptus*; 15♂, 37 km W. of Balledonia, WA, 32.17556S 123.27500E, 18 Mar 1996, J. Forrest, sweeping flowering *Eucalyptus*.

**QM**: 20♂ Weebubbie Cave area, WNW Eucla, WA, 31.65000S 128.76667E, 23 Jan 1987, G. & A. Daniels on *Melaleuca*.

#### Diagnosis.

This species is distinguished from other species in this genus by males with pubescence twice as long on T1 as on T2 and S8 on both sides with conspicuous bristle of dense, thick, long setae. Females with horizontal part of propodeal triangle defined by sharp transverse carina.

#### Description.

*Holotype*. ♂ (SAMA 32-032979), body length 12 mm, head width 3.3 mm.

*Relative head measurements*. HW 50, HL 42, UID 31, UFW 34, LID 28, DMA 30, HVO 2, WOC 17, MOD 3, OOD 7, IAD 8, ASD 3, AOD 8, ML 21, BMW 6, MSL 1, SL 14, SW 3, FL 61,

*Structure*. Head: face longer than wide, clearly converging below; malar space circa 0.16× basal mandibular width; clypeus protuberant, vertex not elevated; gena little wider than 0.7 eye width viewed laterally; flagellum 1.22× head width; flagellomeres 2-5 1.6× as long as wide; flagellomere 11 2.3× as long as wide. Legs not modified: hind tarsus about as long as hind tibia; hind basitarsus 5.5× as long as wide; hind coxa with posterior ventral procession; S7 (Fig. 4C), inner lobes almost straight; S8 (Fig. 4D) bare at apex, both sides with conspicuous bristle of dense thick long setae, apparent in un-dissected specimens; genital capsule (Fig. 4A–B): penis valves progressively broadened from mid of gonocoxite to base of gonostylus.

*Coloration*. Integument black, apart from antenna ventrally, legs orange, apart from femur of front leg and posterior part of femur of middle leg black, tegula brown, marginal zone of T1-6 transparent.

*Sculpture*. Scutum, propodeum and tergites with close pit-reticulation, vertical part of propodeal triangle dull with fine irregular reticulation, horizontal part with radial striae, lateral rim coarsely areolate.

*Pubescence*. Eyes with tiny dispersed hairs. Pubescence orange, apart from posterior margin of T6 and whole of T6-7 black, genae near margin of eye pale orange. Hairs on face erect dense and long, hairs on T1 dense, much longer than on T2-4. Sternites almost bare, apart from S3-4 with narrow dense posterior fringes.

*Paratype*. ♀ (SAMA 32-033292), body length 13 mm, head width 3.8mm.

*Relative head measurements*. HW 50, HL 45, UID 29, UFW 35, LID 30, DMA 29, HVO 2, WOC 16, MOD 3, OOD 6, IAD 8, ASD 3, AOD 9, ML 23, BMW 8, MSL 3, SL 16, SW 3, FL 32.

*Structure*. Head: inner eye margin straight converging below; scape over reaching median ocellus, tapering basally; malar space smooth length about 0.16x width; labial palps reaching beyond apex of extended glossa; maxillary palps reaching beyond apex of extended glossa; clypeus below level of supraclypeus; epistomal suture distinct, almost straight; labrum basally strongly produced, apically flat and smooth; F3–10 length equal to, or less than width, F1 longer than F3; antennal sockets depressed; supraclypeal area strongly raised above frons level; midtibial spur long, robust; inner hind tibial spur with 7 long, slender teeth. Metasoma: marginal zones of tergites with weaker sculpture than on disc; pygidial plate broadly rounded apically, flat, dull; T2-4 almost entirely close punctated.

*Coloration*. Head: integument black with dark brown to black clypeus; mandibles black, brown and smooth medially; scape almost naked, black; flagellum; light brown beneath, dark brown above. Mesosomal integument black. Metasomal integument orange.

*Sculpture*. Scutum and scutellum medially smooth with strong open punctation; metanotum dull and rugose; propodeal triangle with several transverse sharp carinae, basal area narrower than metanotum; Vertex closely and medium size punctated over entire width; clypeus flat, shiny with open large punctures; supraclypeal area polished, flat.

*Pubescence*. Head - hair white, densest on the lower paraocular area; mesosomal hair white; hind basitibial plate with dense, fine hair apparently not branched, not obscuring marginal carina; hind basitarsus with posterior fringe of dense long branched white hair widest anteriorly. Metasomal hair dorsally short, sparse; prepygidial fimbria black; T2-4 with fringes of long white hair.

#### Months collected.

January, March, November.

#### Floral visitations.

*Eucalyptus* (Myrtaceae), *Melaleuca* (Myrtaceae), *Myoporum* (Myoporaceae).

#### Distribution.

Known from seven localities in southern Australia, east and west of the Nullarbor Plain.

#### Etymology.

The species is named after Stan Wann, the grandfather of the co-collector, Beth Tully. Stan Wann grew up in the bush on the north coast of NSW, and had a profound knowledge of the birds and trees in the area.

### Trichocolletes

Taxon classificationAnimaliaHymenopteraColletidae

Cockerell, 1912

#### Remarks.

Ten species of *Trichocolletes* were collected during a Bush Blitz survey at Credo Station circa 120 km NW of Kalgoorlie, Western Australia between 29 August - 9 September 2011. The majority of the *Trichocolletes* species were caught on *Mirbelia microphylla* (Fabaceae) which was profusely flowering at many sites in the reserve. The *Trichocolletes* species *avialis*, *nitens* and *rufibasis* were most common and abundant, but among these also single specimens of *aureotinctus*, *dowerinensis*, *dundanensis* and *leucogenys* were collected. A single male of *Trichocolletes eremophilae* was caught in an area with flowering *Eremophila* (Myoporaceae) and *Senna* (Fabaceae). Additionally, a male (first recorded) and females of *Trichocolletes luteorufus* were collected on Senna glutinosa spp. chatelainiana. Here the male of *Trichocolletes luteorufus* is described.

As we only add the male of a single species, we do not produce a completely new key, but suggest modifying the key for the males presented by Batley and Houston (2013, pg. 5), by inserting couplets as follows:

**Table:** 

32(28)	Hind tibia modified: short and swollen, with plume of long orange hairs (Fig. 7a)	***Trichocolletes luteorufus***
–	Hind tibia not modified	**32a**
32a(32	Length 15–17 mm; S2 with dense, untidy plume	***Trichocolletes marginatus***
–	Length 10–14 mm; sternal pubescence weak	**33**

**Figure 7.**
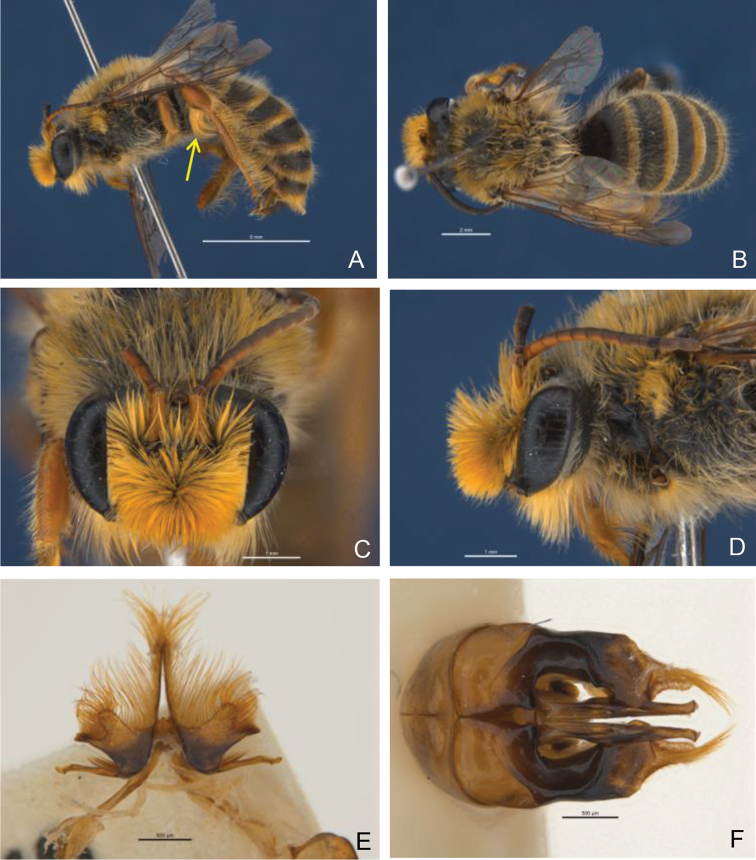
*Trichocolletes luteorufus*. Male (RL1833). **A** lateral **B** dorsal **C** head frontal **D** head lateral **E** S7 **F** genital capsule dorsal.

### Trichocolletes luteorufus

Taxon classificationAnimaliaHymenopteraColletidae

Batley & Houston, 2013

Fig. 7A–F

#### Material examined.

1 ♂ (RL1833), 2 ♀ (RL1834a-b), Credo Station, near Ularring Rock, WA, Australia, 31 Aug 2011, 29.92833S, 120.55209E, R. Leijs, on Senna glutinosa ssp. chatelainiana.

#### Diagnosis.

*Trichocolletes luteorufus* is the only species where the male has distinctly broadened hind tibia bearing a well developed plume of dense long orange hair (Fig. 7A).

#### Description.

♂ (RL1833), body length 15 mm, head width 3.8 mm.

*Relative head measurements*. HW 50, HL 34, UID 33, UFW 33, LID 30, DMA 32, HVO 4, WOC 13, MOD 3, OOD 8, IAD 8, ASD 3, AOD 8, ML 18, BMW 5, MSL 2, SL 12, SW 3, FL 61.

*Structure*. Face wider than long, sligthly converging below; malar space circa 0.4× basal mandibular width; labrum 1/2 as long as wide, convex, roughened by carinae; clypeus protuberant; vertex almost horizontal; gena little wider than 0.5 eye width viewed laterally; flagellum 1.22× headwidth; flagellomeres 4-10 1.67× as long as wide, flagellomere 11 2.1× as long as wide; legs long: hind tarsus 2× as long as hind tibia; hind tibia distinctly broadened medianly (Fig. 7A); hind basitarsus 5× as long as wide; posterior carina of basitibial plate reaches apex; genital capsule (Fig. 7F), inner apex of gonostylus dorsally with broad rectangular lobe reaching penis valve. Penis valve hooked apically; gonostylus laterally with concave vertical plate, inner margin ventrally deeply emarginated (Fig. 7F); S7 (Fig. 7E) lateral lobe, emarginated, at basis near ligulate process carrying 4 long stiff setae; posterior projection very long.

*Coloration*. Integument black, apart from scape, antenna ventrally and all legs from posterior part of femur onwards orange; marginal zone of tergites translucent pale orange; sternites brown; labrum and basal 2/3 of mandible transparent white, mandible apex brown.

*Sculpture*. Vertex and scutum with fine transverse lineo-reticulation and open to sparse punctation.

*Pubescence*. Eyes with tiny dispersed hairs. Face, especially clypeus, with very dense, long, orange hair, finely-branched on clypeus and inner eye margins; hair on vertex more open, fine and white. Ventral part of gena with dense long ventrally directed branched pale orange hair, outer eye margins with short white hair. Scutum with medium length pale orange hairs, remaining part of thorax with off-white hairs. Fore basitarsus with plume of orange hair; hind tibia with distinctive plume of dense long orange hair (Fig. 7A); inner area of hind tibia and basitarsi with long dispersed bended orange hairs. T1-3 openly covered with long, erect, white hair, decreasing in length; S3-5 with long bended white to orange hairs that together almost forms a corbicula like structure.

#### Remarks.

Previously the male of *Trichocolletes luteorufus* was unknown (Batley and Houston 2012). The sexes were linked based on the fact that both males and females were collected on the same flowering bush of Senna glutinosa subsp. chatelainiana, where males were chasing females of this species.

#### Distribution.

Known from two localities, near Mt Magnet and Credo Station, North of Coolgardie, WA.

## Discussion

The Australian native bee fauna is as yet not completely known, and many new species are added with each new generic revision (Batley and Hogendoorn 2009). Here we have shown that surveying remote locations in Australia can even turn up new species in genera that have only recently been revised. This emphasizes the fact that our knowledge to date remains fragmentary.

## Supplementary Material

XML Treatment for Goniocolletes

XML Treatment for Goniocolletes comatus

XML Treatment for Goniocolletes wanni

XML Treatment for Trichocolletes

XML Treatment for Trichocolletes luteorufus
